# Attraction of the Larval Parasitoid *Spintherus dubius* (Hymenoptera: Pteromalidae) to Feces Volatiles from the Adult *Apion* Weevil Host

**DOI:** 10.1007/s10905-017-9605-5

**Published:** 2017-02-08

**Authors:** N. Faraone, G. P. Svensson, O. Anderbrant

**Affiliations:** Department of Biology, Lund University, Sölvegatan 37, SE-223 62 Lund, Sweden

**Keywords:** Larval parasitoid, oviposition cues, kairomone, host feces, *Apion trifolii*, *Apion fulvipes*

## Abstract

The behavioral response of the larval parasitoid *Spintherus dubius* (Hymenoptera: Pteromalidae) to volatile compounds derived from its *Apion* weevil hosts was investigated in two-choice bioassays. Odor source candidates were the larval and adult stages of weevils, clover flowers, and feces from adult weevils. Despite *S. dubius* being a larval parasitoid, the odor of weevil larvae isolated from the clover flowers was not attractive to female parasitoids. Surprisingly, *S. dubius* females were instead attracted by the odor from the feces of adult weevils. The female parasitoids were similarly attracted to the feces produced by the two main hosts, the red clover weevil (*A. trifolii*) and the white clover weevil (*A. fulvipes*)*.* Chemical analysis of the volatile composition of feces produced by the two hosts revealed qualitatively similar odor profiles, correlating with the observed attraction by the parasitoid towards both odor sources. Some of the identified volatile compounds are commonly present in clover plant headspace fractions and may function as a kairomone to facilitate orientation by *S. dubius* to *Apion*-infested clover flowers. Larval and adult weevils were not attractive for parasitoid females, whereas, for the white clover weevil-plant association, infested flowers were highly attractive. These data show the use by the clover weevil parasitoid of an alternative source of olfactory information for locating its host.

## Introduction

Insect herbivores as well as their natural enemies rely heavily on olfactory cues for locating food and oviposition sites, even at great distances. In the case of parasitoids, volatiles from the insect host itself (Cournoyer and Boivin 2004; Chuche et al. 2006; Obeysekara and Legrand 2014), from the host habitat (Rutledge 1996), from sources indirectly related to the host (Steidle and Schöller 1997; Sullivan and Berisford 2004) or their combination with plant volatiles released during herbivore attack (Obeysekara et al. 2014; Wäschke et al. 2014) may contribute to host localization. Moreover parasitoids may use different chemical volatiles emitted from different sources in various stages of the host location process (Rutledge 1996).

*Spintherus dubius* (Nees, 1834), a small hymenopteran wasp (2 mm long), is the most common parasitoid species found in clover fields (Kruess and Tscharntke 1994; Lundin 2013), and the main hosts are weevils of the genus *Apion*, which are important pests in clover seed production (Hansen and Boelt 2008). In Sweden, *S. dubius* mainly attacks *A. fulvipes* (Geoffry, 1785) feeding on white clover, *Trifolium repens* L., and *A. trifolii* (L., 1768) feeding on red clover, *T. pratense* L. (Gønget 1997). These weevils overwinter below dead leaves or grass, and in the spring overwintered adults emerge and locate host plants where they feed and mate (Freeman 1967). Mature weevils start to mate during the early flowering phenology of the clover host, and females oviposit in the developing clover head. The weevil larvae feed on the clover seeds and they stay inside the floret/flower head during all their development (Notini 1935; Kruess and Tscharntke 1994; Nyabuga et al. 2015). They pupate inside the flower, and only when they reach their adult stage, they leave the flowers and emerge as young weevils.

**Fig. 1:**
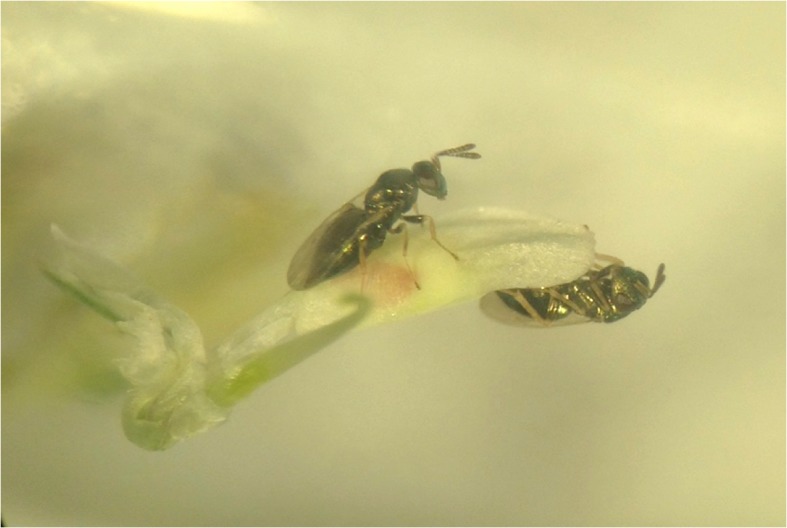
Host-location process of *Spintherus dubius* females on infested white clover flowers (*Trifolium repens*). Photo by Nicoletta Faraone

After mating, female *S. dubius* are observed walking around on the clover flowers, drumming the florets with the antennae (i.e. antennation) as for searching cues for localizing the host (personal observation) (Fig. 1). Once located, females deposit an egg inside the weevil larva (medium to late instar), and the parasitoid larva feeds on the host larval body causing its death as a final result. The host location ability seems being highly developed in the female parasitoid that is able to detect the presence of a weevil larva, even when surrounded by a very complex odor background (Kigathi et al. 2009; de Rijk et al. 2016).

Whether *S. dubius* uses olfactory cues in the host location and recognition processes has not been investigated. In the present study we aimed to identify the source of olfactory cues that the female parasitoid uses for locating its *Apion* host in infested clover flowers. The selection of the odor stimuli was made based on the most potential candidate sources for host location cues and odors that characterized the common background bouquet of a clover field. Potential odor sources were volatiles from clover flowers, from larvae and adult weevils, and from feces of adult weevils, and we used them in bioassays to evaluate the response of *S. dubius* females to different olfactory cues.

## Materials and Methods

### Insects and Plants

Adult *S. dubius* were collected by using a sweeping net in organic fields of red clover and white clover in August–September 2015 and 2016, in Skåne province, Sweden. In addition, when in full bloom, infested clover flowers were collected from these fields and placed in Plexiglas® cages and cardboard tubes (40 cm × 10 cm i.d.) under laboratory conditions (20 ± 2 °C; 70 ± 2% RH; 16:8, L:D) where *S. dubius* emerged a few days later. Wasps collected from the field or newly emerged wasps from infested flowers were transferred to small plastic boxes with sugar-water solution (1:1) and kept under the same laboratory conditions. Males and females were kept in the same boxes to allow them to mate. All parasitoids used for behavioral bioassays were 2–5 days old females that were presumably mated. No differentiation was done between females with or without experience in oviposition. Because *S. dubius* adults do not survive more than one week under laboratory conditions, field collection of insects continued until the completion of the experiments.

*A. fulvipes* and *A. trifolii* adults were kept under the same lab conditions as the wasps for feces collection. Weevils were collected from the same location as the parasitoids with the same sweeping technique and kept in Plexiglas® cages. Weevil feces were scraped off from the surfaces of the cages using a spatula and directly used for the two-choice bioassay (25 mg of feces used for each test as stimulus). Previously planted red (variety SW Nancy) and white (variety Hebe) clover plants, located in a common garden under natural conditions (Dept. Biology, Lund University, Sweden), were used for non-infested floral tissue collection. Infested clover flowers were collected from red (variety SW Nancy) and white clover (varieties Undrom and Bombus C Krav) organic fields (Skåne province, Sweden).

### Behavioral Experiments

**Fig. 2:**
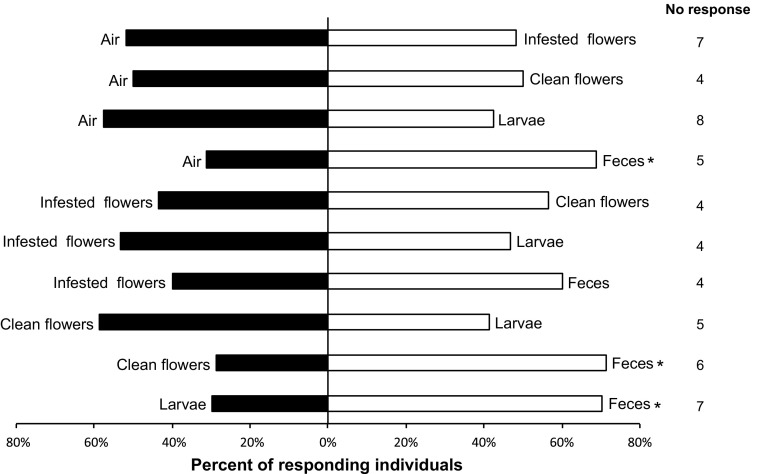
Response of individual walking *Spintherus dubius* females from red clover (*Trifolium pratense*) in a Y-tube olfactometer to different odor stimuli. Feces were collected from *Apion trifolii* weevils. For each pair of stimuli, 34 insects were tested. Bars denoted by asterisk indicate a significant preference for the treatment (* *P* < 0.05)

**Fig. 3:**
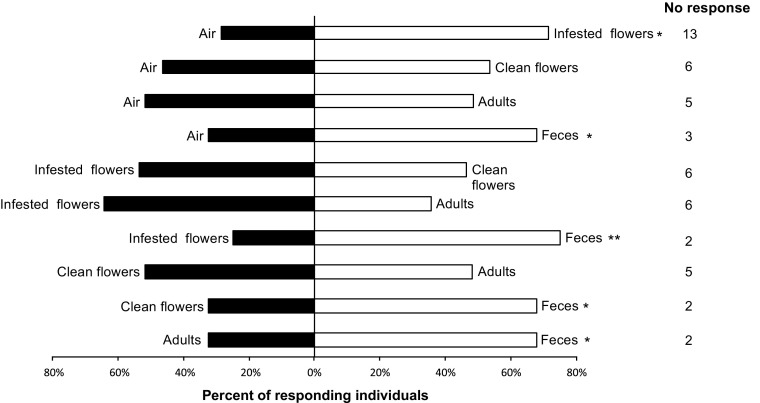
Response of individual walking *Spintherus dubius* females from white clover (*Trifolium repens*) in a Y-tube olfactometer to different odor stimuli. Feces were collected from *Apion fulvipes* weevils. For each pair of stimuli, 34 insects were tested. Bars denoted by asterisk indicate a significant preference for the treatment (* *P* < 0.05, ** *P* < 0.01)

*S. dubius* females were subjected to 10 binary choice tests for each weevil-plant association (Figs. 2,3). Odor sources were changed after each experimental set. On each day of trial, pairs of stimuli were randomly offered to the test insect, and for each new pair of stimuli a new insect was used (*n* = 34). Prior to starting the experiments, selected clover flowers and buds were examined under a microscope for detecting residual presence of feces, piercing holes in the florets and traces of previous presence of the weevils. These floral tissues were accordingly classified as either infested or non-infested. Clean/non-infested flowers/buds were washed gently with distilled water and left to dry before using them as odor sources. Larvae (medium-to-late instars) of *A. trifolii* were removed from infested red clover flowers and used as odor sources (5 larvae per test). Because larvae from *A. fulvipes* were smaller and difficult to remove from the white clover florets, and because our previous experiments showed that female parasitoids were not attracted by *A. trifolii* weevil larvae, adult weevils of *A. fulvipes* (10 female and 10 males) were selected as odor stimulus instead.

The behavioral response of *S. dubius* females to various odor sources was tested in a two-choice olfactometer, similar in design and operation to that of Sullivan et al. (2000). Each arm of a Pyrex glass Y-tube (5 mm i.d.; stem 80 mm; arms 50 mm at 135° angle to the stem) was attached with Teflon tubing to a sealed glass source chamber (internal volume 30 ml). Air that entered the glass source chamber was charcoal-filtered and humidified, and the airflow through each arm of the Y-tube was maintained at 20 cm s^−1^ (150 ml min^−1^). The flow was controlled by a flow-meter (Porter, Hatfield, PA, USA). One hour prior to the experiments, female parasitoids were transferred individually to glass test tubes covered with a cotton cup and placed in the bioassay room for acclimatization. During experiments, the bioassay room was held at 23 ± 2 °C and light was provided only by a diffused strip light array centered over the Y-tube, that was positioned on a white table. Trials were performed during the photophase hours (between 10:00 and 18:00). A female parasitoid was placed into the opening of the stem of the Y-tube and it was observed for a maximum of 5 min. Individual females were recorded as choosing a given arm of the Y-tube if they spent more than 15 s beyond a line positioned 3 cm upwind from the Y-intersection. Parasitoids that failed to make a choice within the given time period were considered as non-responders. The assignment of odor sources to each arm of the olfactometer was reversed after every pair of stimuli test to eliminate any directional bias. Parasitoids were discarded after each test, and the Y-tube was replaced with a clean one after every second trial. After use, the Pyrex glass equipment (Y-tubes and glass source chambers) were washed with odor-free dishwashing detergent and 70% ethanol, and then dried in the oven at 110 °C for 1 h.

The null hypothesis that the female parasitoid showed no preferences for either odor stimulus in a two-choice experiment (i.e., the hypothesized choice proportion was 50:50) was tested with *χ* ^*2*^ analyses (Microsoft Excel 2011).

### Collection and Analysis of Volatiles

Feces from *Apion* weevils were collected with a spatula from the Plexiglas® cages where adult weevils were kept under conditions described above, and then placed in a 2 ml vial with a rubber septum lid. Headspace collection was performed using small Teflon filters (3 mm i.d.), filled with 30 mg of Super-Q adsorbent (Alltech, PA, USA), inserted through the lid and connected to a battery-driven air pump (GroTech, Gothenborg, Sweden). The flow rate of the pump was set to 250 ml min^−1^. After 6 h of sampling, the volatiles were eluted from the traps with 150 μl of n-heptane containing an internal standard (tetradecane, Sigma-Aldrich) at a concentration of 3 ng μl^−1^. Three collections were performed for each weevil species, and each vial containing the feces was matched with an empty vial as a control to identify potential background contaminants. Headspace samples were analyzed with an Agilent 7890A gas chromatograph, equipped with a non-polar HP-5MS capillary column (30 m × 0.25 mm, film thickness 0.25 μm, J&W Scientific, USA), linked to an Agilent 5975C mass spectrometer. Helium was used as carrier gas at a velocity of 40 cm sec^−1^, and the injector temperature was 250 °C. Oven temperature was programmed from 50 °C (held for 1 min) to 240 °C at 8 °C min^−1^, and then held at 240 °C for 10 min. Compounds were identified by using mass spectral libraries, and by comparison of Kovats retention index, retention times and mass spectra with those of synthetic standards.

## Results

### Behavioral Experiments

In the olfactometer, the female parasitoids either showed grooming or walking behavior. When comparing the different treatments, we observed significant differences between odor stimuli and particularly female parasitoids were attracted by chemical cues released from the adult weevil feces. When tested against clean air, female parasitoids were significantly attracted to volatiles from *A. trifolii* feces (*N* = 29; *χ* ^*2*^ = 4.2; *df* = 1; *P* < 0.05; Fig. 2) and from *A. fulvipes* feces (*N* = 31; *χ* ^*2*^ = 3.9; *df* = 1; *P* < 0.05; Fig. 3). In experiments associated with the *A. trifolii*-*T. pratense* complex, significant attraction of female parasitoids towards the feces was also confirmed when they were tested against weevil larvae (*N* = 27; *χ* ^*2*^ = 4.5; *df* = 1; *P* < 0.05), or against non-infested clover flowers (*N* = 28; *χ* ^*2*^ = 5.1; *df* = 1; *P* < 0.05). No significant preference was shown by *S. dubius* females when offered other pairs of stimuli. Interestingly, in experiments associated with the *A. fulvipes*-*T. repens* complex, a significant attraction towards the weevil feces was recorded when that stimulus was paired with the odor from infested clover flowers (*N* = 32; *χ* ^*2*^ = 8; *df* = 1; *P* < 0.01). A significant preference for the weevil feces was also observed when offered in combination with non-infested clover flowers (*N* = 31; *χ* ^*2*^ = 3.9; *df* = 1; *P* < 0.05), or with adult weevils (*N* = 31; *χ* ^*2*^ = 3.9; *df* = 1; *P* < 0.05). Female parasitoids were also significantly attracted by infested white clover flowers when paired with clean air (*N* = 21; *χ* ^*2*^ = 3.9; *df* = 1; *P* < 0.05), which was in contrast to the lack of such preference in the experiment performed with infested red clover flowers versus clean air. The contrasting results may be explained by different amount of feces (impossible to quantify) deposited on the floral tissue of the two host plants used as stimuli in the bioassays. No preference by *S. dubius* females was observed in tests including other pairs of stimuli for the *A. fulvipes*-*T. repens* complex.

### Chemical Analysis

**Fig. 4:**
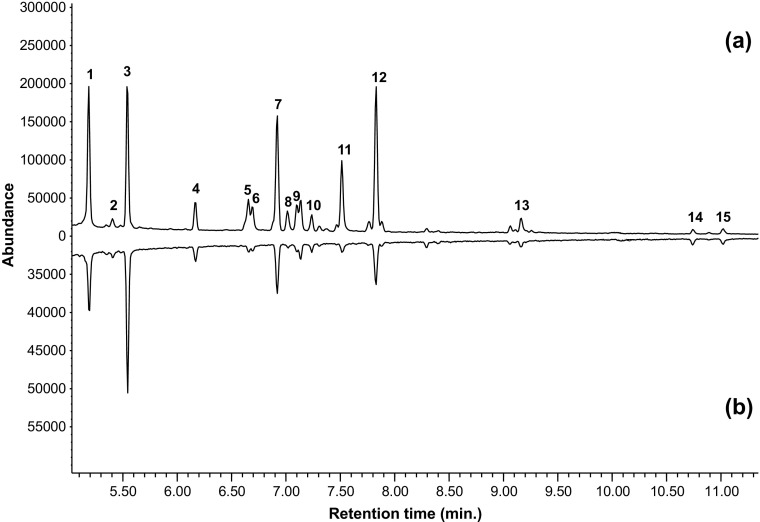
Composition of headspace volatiles emitted by *Apion fulvipes* feces (**a**) and *Apion trifolii* feces (**b**) after 6 h of collection and analyzed by gas chromatography. Note the different scales on the y-axes for the two species. Numbers refer to compounds listed in Table 1

A comparison of the chemical profiles of feces from the two weevil species revealed the same set of compounds produced by the different hosts. In total 15 volatiles were consistently detected in the feces samples of both *A. trifolii* and *A. fulvipes* adults but not found in the control samples (Fig. 4, Table 1). The analysis revealed volatiles commonly found in clover plants, including terpenes (i.e. alpha- and beta-pinene, limonene), aldehydes (i.e. nonanal and decanal) and compounds belonging to the ‘green leaf volatile’ group (i.e. (*E*)-2-hexenyl acetate and 4-methyl-3-heptanol). Two ketones were also identified (2-heptanone and 6-methyl-5-hepten-2-one). Major compounds were in both species represented by isoamyl acetate, nonane, limonene and beta-pinene.

**Table 1:** Composition of headspace volatiles found in the headspace samples of feces from adult *Apion trifolii* and *Apion fulvipes*

^a^ID	RT (min.)	RI	^b^Compound	^c^Standards	^d^ *A. trifolii*	^d^ *A. fulvipes*
1	5.179	877	Isoamyl acetate	Sigma-Aldrich	0.065 ± 0.027	0.084 ± 0.058
2	5.404	903	2-Heptanone	Sigma-Aldrich	0.015 ± 0.015	0.014 ± 0.009
3	5.546	911	Nonane	Sigma-Aldrich	0.203 ± 0.061	0.275 ± 0.216
4	6.136	945	α-Pinene	^e^KTH	0.029 ± 0.008	0.028 ± 0.007
5	6.654	970	4-Methyl-3-heptanol (threo)	^f^WSU	0.007 ± 0.006	0.020 ± 0.020
6	6.688	974	4-Methyl-3-heptanol (erythro)	^f^WSU	0.006 ± 0.006	0.012 ± 0.012
7	6.913	990	β-Pinene	Sigma-Aldrich	0.086 ± 0.017	0.096 ± 0.043
8	7.013	992	6-Methyl-5-hepten-2-one	^g^SLU	0.003 ± 0.003	0.012 ± 0.012
9	7.096	997	Myrcene	Sigma-Aldrich	0.017 ± 0.004	0.019 ± 0.013
10	7.238	1000	Decane	Sigma-Aldrich	0.009 ± 0.002	0.015 ± 0.004
11	7.513	1018	(*E*)-2-Hexenyl acetate	Sigma-Aldrich	0.012 ± 0.007	0.034 ± 0.030
12	7.830	1035	Limonene	Sigma-Aldrich	0.077 ± 0.019	0.102 ± 0.061
13	9.164	1104	Nonanal	Aldrich-chemi	0.005 ± 0.004	0.009 ± 0.009
14	10.739	1191	Methyl salicylate	Sigma-Aldrich	0.015 ± 0.004	0.008 ± 0.003
15	11.031	1204	Decanal	Aldrich-chemi	0.004 ± 0.004	0.004 ± 0.004

*RT* = Retention time. *RI* = Retention index

^a^In order of elution during gas chromatography

^b^The identity of all compounds was confirmed by NIST library and comparison with synthetic standards and Kovats retention index

^c^Overall purity 95–99%

^d^Mean ± SE quantity (ng) volatiles collected after 6 h from 10 mg of raw feces (*N* = 3). Quantification was performed using an internal standard at the concentration of 3.1 ng μl^−1^

^e^Gift from A.-K. Borg-Karlson, Royal Institute of Technology, Stockholm, Sweden

^f^Synthesized by D.S. Matteson, Washington State University, Pullman, USA (Anderbrant et al. 2010)

^g^Gift from G. Birgersson, Swedish University of Agricultural Sciences, Alnarp, Sweden

## Discussion

In this study we have identified a new mechanism by which a natural enemy can exploit olfactory information derived from its host. Our experiments show that volatiles released from feces of adult *Apion* weevils function as a kairomone to attract females of their larval parasitoid *S. dubius*. To our knowledge, this is the first study to report that volatiles released from feces of the adult stage of the host species can attract females of a larval parasitoid, thereby acting as an indirect signal for host detection and location. Surprisingly, the female wasps were not attracted by the odor of *A. trifolii* larvae, i.e. the substrate in which they lay their eggs. Feces from host larvae were not part of the test panel of stimuli in this study, but we predict that the feces from the adult weevils (that primarily feed on leaves) and the weevil larvae are similar in chemical composition. Indeed, mid-to-late instar *Apion* larvae do not only feed on clover seeds but also on the floret and its surroundings (Lundin 2013). The use of feces cues for detecting the final target is not uncommon in parasitoids, and other species (such as larval and adult parasitoids, respectively) have been reported to use the volatiles released from larval (Chuche et al. 2006) and adult feces (Meiners and Hilker 1997) for host location.

Although herbivore-induced plant volatiles (HIPVs) can be a useful cue acting as a kairomone/synomone for the parasitoid during host search, feeding by other insect species than its own host may cause similar damage on the plants, inducing the release of a similar blend of volatiles (de Rijk et al. 2016). In such cases, the olfactory signal provides insufficient information to the parasitoid regarding the identity of the herbivore, and it may therefore need more reliable cues, such as specific chemical volatiles that are directly produced by the host. In addition, non-host herbivores may interfere with the odor-mediated location of host-infested plants by the parasitoid and reduce encounter rates with the hosts on the plant. For this reason feces represent a reliable cue for the parasitoid (Sullivan et al. 2000; de Rijk et al. 2016). In fact, once the parasitoids find the location of host-associated plants and land on them, herbivore host-specific odors become critical short-range cues in the last phase of the host location process (Rutledge 1996).

The identification of the specific blend of HIPVs that elicits the attraction of a parasitoid can be difficult but represents a key step in understanding many plant-herbivore-parasitoid tritrophic interactions. For instance, the combination of volatiles from the insect host and the plant it is feeding on may act as a synergistic signal used by the female parasitoid for host selection and location (Guerra et al. 1994). Volatiles from feces can be a hint for locating the weevil larva, but it might be possible that the female parasitoid also uses close-range volatiles from the plants for orientation (i.e. egg-induced plant cues) in order to get to the larva inside the floret (Hilker and Fatouros 2015). The similar composition of feces headspace samples from *A. fulvipes* and *A. trifolii* shows that the female parasitoids probably use the same set of volatiles in host search in both cases. The source of food for the host has a crucial role in determining the nature of chemical cues emanating from them, and they might be essential in defining the status of the host and if it is at the suitable stage for being parasitized (Steidle and Van Loon 2003).

The foraging activity of natural enemies of insects relies on a rich variety of semiochemicals from a host-plant complex. However, only a subset of the host-associated volatiles may be attractive or ecologically relevant to parasitoids for the final host location and recognition (Dweck et al. 2010). The challenge is to understand how the blend of volatiles is perceived by natural enemies (i.e. as single volatile component or as a mixture) and if a specific ratio of volatiles triggers the attractant response (Clavijo McCormick et al. 2014). Although attraction of parasitoids elicited by the volatiles from the feces was observed, we do not know yet which specific compounds (or mix of compounds) are responsible for the attraction and trigger the orientation towards the host. Recent studies have suggested that there is redundancy in the composition of host odor blends, and most likely not all compounds released are essential for host recognition and identification (Nojima et al. 2003; Morawo and Fadamiro 2016). On the other hand, there may be more unselective acceptance of host odor patterns with recognition of several host odor blends (Tasin et al. 2007; Bruce and Pickett 2011). Valuable information can be extracted from these observations because the chemical classes of the volatiles responsible for the parasitoid attraction/orientation can be an indicator of the host-utilization stage. Terpenoids (such as alpha- and beta-pinene, limonene), and aldehydes (such as decanal and nonanal) found in the weevil feces headspace collections are predominantly found in the habitat-location stage as synomones, but there are examples where they were kairomones (Rutledge 1996). Moreover, many of the alcohols, aldehydes and esters, identified as synomones in the host location stage, belong to the ‘green leaf volatiles’ class (e.g. trans-2-hexenyl acetate) (Whitmann and Eller 1992), which are commonly found in the surrounding area of both damaged and undamaged plants. Regarding compounds associated with the host acceptance and oviposition, ketones represent a class of compounds commonly found in host feces (Ramachandran et al. 1991). Therefore, several classes of compounds are candidates as semiochemicals used by *S. dubius* females in the host location process.

This work is a first step toward understanding the chemically-mediated interaction between *S. dubius* and its two main hosts, *A. fulvipes* and *A. trifolii*, and in these initial experiments we focused on the behavioral response of the parasitoid to natural odor sources. We found consistent attraction of female wasps to volatiles released from the host feces, not considering which chemical compound (or blend of compounds) can be essential for the female wasp in its host recognition and acceptance. The next steps will be to identify the active compound(s) in the host feces, and test these in bioassays. The small size of the *S. dubius* antenna makes standard screening for active compounds with gas chromatographic-electroantennographic detection very difficult, but single sensillum recordings can hopefully be applied to analyze the physiological response to individual host feces volatiles. Such analyses will then be complemented with behavioral tests in the laboratory and in the field to fully understand the kairomonal response of *S. dubius* during host search.
